# Bacteremia (Sepsis), Hepatorenal Syndrome, and Serum Creatinine Levels Rather than Types or Microbial Patterns Predicted the Short-Term Survival of Cirrhotic Patients Complicated with Spontaneous Bacterial Peritonitis

**DOI:** 10.3390/diagnostics13010094

**Published:** 2022-12-28

**Authors:** Chien-Hao Huang, Sheng-Fu Wang, Chen-Hung Lee, Yen-Mu Wu, Ching Chang, Bo-Huan Chen, Yu-Tung Huang, Yu-Pin Ho

**Affiliations:** 1 Division of Hepatology, Department of Gastroenterology and Hepatology, Chang-Gung Memorial Hospital, Linkou Medical Center, Taoyuan 33305, Taiwan; 2 College of Medicine, Chang-Gung University, Taoyuan 33302, Taiwan; 3 Division of Cardiology, Department of Internal Medicine, Chang Gung Memorial Hospital-Linkou, Taoyuan 33305, Taiwan; 4 Department of Infectious Disease, Chang-Gung Memorial Hospital, Linkou Medical Center, Taoyuan 33305, Taiwan; 5 Center for Big Data Analytics and Statistics, Department of Medical Research and Development, Chang Gung Memorial Hospital, Linkou Medical Center, Taoyuan 33305, Taiwan

**Keywords:** spontaneous bacterial peritonitis and types, liver cirrhosis, extended-spectrum beta-lactamases resistant (ESBL), Gram-positive bacteria (GPC), bacteremia or sepsis, hepatorenal syndrome (HRS)

## Abstract

(1) Background: Spontaneous bacterial peritonitis (SBP) is a major and severe complication in cirrhosis patients with ascites. Over the years, advance in antibiotic treatment has led to changes in microbial patterns in some regions, including the emergence of extended-spectrum beta-lactamases resistant (ESBL)-producing bacteria and an increase in Gram-positive bacteria (GPC). In addition, three SBP types (classic SBP, culture-negative neutrophilic ascites (CNNA), and monomicrobial non-neutrocytic bacterascites (MNB)), may also have different prognoses. Therefore, the study aimed to investigate the microbial pattern and the predictors of short-term outcomes in patients with SBP. (2) Methods: Patients discharged with a diagnosis of the first episode of SBP between January 2006 and July 2017 were enrolled. Patients’ clinical, demographic, hematological, and biochemical data were obtained at diagnosis, and the model for end-stage liver disease (MELD)-based scores were calculated accordingly. Patients were followed up until February 2018 or until death. (3) Results: A total of 327 patients were analyzed. The prevalence of classic SBP was nearly equivalent to CNNA. As for the microbial pattern, Gram-negative bacillus (GNB) remained more prevalent than GPC (75 vs. 25%), with *E. coli* being the most common bacterial species, followed by *K. Pneumoniae* and then *Staphylococcus*. The percentage of ESBL strain in culture-positive patients was 10.9%. By univariable and multivariable logistic regression survival analysis, there was no significant difference in predicting short-term mortality among the three SBP types, neither between GNB vs. GPC nor between ESBL- and non-ESBL-producing bacteria. Only bacteremia (sepsis), hepatorenal syndrome (HRS), and serum creatinine (Cr) were independent predictors of in-hospital and 3-month mortality, whereas HRS and Cr were independent predictors of 6-month mortality. (4) Conclusions: SBP types, Gram stain result, and ESBL strain did not affect survival. Only bacteremia (sepsis), HRS, and serum Cr independently predicted the short-term mortality in patients with SBP.

## 1. Introduction

Cirrhotic patients have an impaired defense system against bacteria associated with reduced bacterial clearance [1,2]. This immune defect facilitates bacterial translocation induced by increased intestinal permeability and intestinal bacterial overgrowth observed in cirrhosis [3]. Bacterial infection is present at admission or develops during hospitalization in about 30% of patients with cirrhosis [4]. The most common infection was spontaneous bacterial peritonitis (SBP) [4].

SBP is a serious complication representing an advanced stage in patients with cirrhosis and ascites [5,6]. There are three types of SBP: (1) classic SBP; (2) culture-negative neutrophilic ascites (CNNA); and (3) monomicrobial non-neutrocytic bacterascites (MNB). The 2013 American Association for the Study of Liver Diseases (AASLD) guideline on the management of adult patients with ascites due to cirrhosis suggested empiric antibiotic therapy for patients with ascites fluid PMN counts ≧ 250 cells/mm^3^ or <250 cells/mm^3^ but with symptom/sign of infection [7]. This implies that all three types of SBP need prompt treatment when a symptom/sign of infection is present. In addition, when first reported, the in-hospital mortality of an episode of SBP exceeded 90%; however, the rate has been reduced to approximately 20% through early diagnosis and prompt antibiotic therapy [8]. Nevertheless, the prognosis of these three types of SBP is rarely compared with each other.

Bacterial translocation from the gastrointestinal tract is the most common source of SBP. Thus, two-thirds of SBP cases were caused by GNB, of which *Escherichia coli* is the most frequently isolated pathogen [4,8]. Therefore, the 2013 AASLD guideline and 2018 EASL guideline recommended that the first-line antibiotic treatment for SBP is third generation cephalosporins [7,9]. However, changes in the patterns and microbiology of SBP have been observed in some regions over the past few years, such as the increased prevalence of CNNA, ESBL-producing bacteria, increased resistance rate to first-line antibiotics [10], and higher frequency of Gram-positive organisms [11]. In a Netherlands report, a nonsignificant increase in the proportion of patients with SBP caused by Gram-positive bacteria and multidrug-antibiotic-resistant bacteria over 10 years was found [12], prompting our interest in studying the microbial pattern of SBP and whether it would influence the prognosis.

Therefore, the study aimed to investigate the incidence and prognosis of the three types of SBP, the microbial pattern, and to determine whether the form, the bacterial pattern, and the drug-resistant strain would influence the prognosis of SBP.

## 2. Materials and Methods

### 2.1. Patient Selection and Follow-Ups

With the approval of the ethical committees of Chang Gung Memorial Hospital (202000112B0), a list of patients with a discharge diagnosis of SBP and liver cirrhosis between January 2006 and July 2017 was obtained sequentially from the medical record management committee. A total of 327 patients who met the criteria for SBP and were diagnosed for the first time were included in the retrospective study. Patients’ clinical, demographic, hematological, and biochemical data were obtained at diagnosis, and the MELD-based scores were calculated accordingly. Patients were followed up until February 2018 or until death.

### 2.2. Diagnosis, Definition, and Management of Liver Cirrhosis and Spontaneous Bacterial Peritonitis

The diagnosis of liver cirrhosis was based mainly on the following criteria: (1) Typical sonographic diagnosis for liver cirrhosis [13]; (2) ascites were caused by liver cirrhosis (serum-ascites albumin gradient > 1.1 g/dL) [14]; (3) exclusion of other underlying diseases such as malignancy (HCC or metastasis), right-sided congestive heart failure, Budd–Chiari syndrome, post-sinusoidal obstruction syndrome, portal or splenic vein thrombosis, and the possibility of schistosomiasis. Management of liver cirrhosis was in accordance with the AASLD, Baveno VI, as well as the Asian Pacific Association for the Study of the Liver (APASL) guidelines [7,15,16].

SBP was diagnosed upon positive ascites culture and/or an absolute neutrophil count in ascites fluid of ≧250 cells/mm^3^, in the absence of a surgically treatable source of infection and other causes of elevated ascites neutrophil count, such as hemorrhage, pancreatitis, peritoneal tuberculosis, or carcinomatosis [17,18,19]. The treatment of SBP adhered to the recommendations of the International Ascites Club and AASLD guidelines [17].

There are three variants of SBP: (1) classic SBP (elevated PMN count >250/mm^3^ and positive culture); (2) culture-negative neutrophilic ascites (CNNA): ascites culture is negative and PMN cell count is >250/mm^3^ (3) monomicrobial non-neutrocytic bacterascites (MNB), in which PMN count < 250/mm^3^ but the culture was positive.

### 2.3. The Outcomes of SBP

Due to the high short-term mortality, the outcome or prognosis of SBP was defined as the in-hospital, 3-month (3 M), and 6-month (6 M) mortality.

### 2.4. The Diagnosis of Hepatorenal Syndrome and Hepatic Encephalopathy

The hepatorenal syndrome was diagnosed based on clinical criteria brought up by the AASLD, the International Club of Ascites (ICA), and Kidney Disease: Improving Global Outcomes (KDIGO) [20,21]. The prerequisites were the absence of any other apparent cause for the acute kidney injury, including shock, current or recent treatment with nephrotoxic drugs, and the absence of ultrasonographic evidence of obstruction or parenchymal kidney disease.

Hepatic encephalopathy (HE) was diagnosed according to the European Association for the Study of the Liver (EASL)/American Association for the Study of Liver Diseases (AASLD) guidelines [22,23] and by the exclusion of other causes of mental status changes. HE was graded by the West Haven Criteria [24].

### 2.5. The Diagnosis of Bacteremia (Sepsis)

Bacteremia was diagnosed when at least two serial sets of blood cultures yielded positive bacteria species. Sepsis was defined as two or more SIRS criteria with documented infection focus [25] (SBP in this study).

### 2.6. Calculations of Predicting Scores

The MELD score was 11.2 × ln (international normalized ratio (INR)) + 9.57 × ln (creatinine, mg/dL) + 3.78 × ln (bilirubin, mg/dL) + 6.43) with lower bound of one for all three variables and an upper bound of four for serum creatinine. The MELD-Na score was MELD score − Na − (0.025 × MELD score × (140 − Na)) + 140, in which Na was bounded at 125 and 140. The iMELD score was MELD score + (Age × 0.3) − (0.7 × Na + 100) [26].

### 2.7. Methods/Assays Used For Serum Biochemistry and Hemogram

The method/assays used for serum biochemistry were as follows: serum creatinine: colorimetry (reference value: F:0.44–1.03, M: 0.64–1.28 mg/dL); serum bilirubin: spectrophotometry (reference value:0.3–1.2 for <60 y/o, 0.2–1.1 for 60–90 y/o, 0.2–0.9 for >90 y/o mg/dL); serum AST/ALT: enzymatic method (reference value: AST: ≦34 U/L, ALT ≦ 36 U/L); serum Na: ion-selective sensor (reference value: 136–146 mEq/L); serum albumin: colorimetry (reference value: 3.5–4.5 g/dL), respectively.

The method/assays used for serum hemogram were as follows: serum INR: electrochemical method (reference value: 2.0–3.0); WBC and PLT: automated cell count (reference value: WBC: 3.9–10.6 10^3^/μL; PLT: 150–400 10^3^/μL); Hb: spectrophotometric method (reference value: M:13.5–17.5; F:12–16 g/dL), respectively.

### 2.8. Statistical Analysis

Continuous variables were expressed as mean ± standard deviation (SD) or median and interquartile range (IQR, 25–75 percentile), depending on their distribution. Non-parametric Kruskal Wallis test or Mann–Whitney U test was used to compare continuous variables among three groups, or between two groups respectively. Post-hoc tests including Bonferroni correction were used to adjust for the significance level for multiple pairwise comparisons and multiple testing correction. Categorical variables were reported as frequencies or counts with percentages. Their significance was calculated by Chi-square test first while Fisher’s exact test was performed instead when more than 20% of the cells have expected frequencies less than 5. Survival analyses were performed by univariable and/or multivariable logistic regression analysis.

Statistics were performed using SPSS software (SPSS Inc., Chicago, IL, USA, Version 22). A *p*-value of <0.05 was considered statistically significant.

## 3. Results

### 3.1. Flowchart

**Figure 1:**
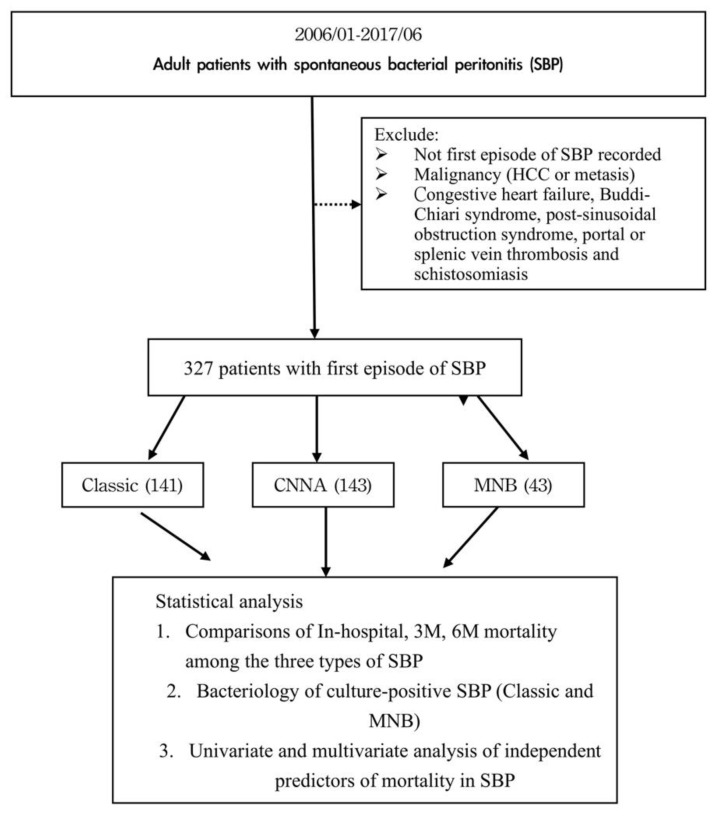
Flow chart.

As delineated in Figure 1, a total of 327 patients diagnosed with the first episode of SBP were enrolled after inclusion and exclusion criteria.

### 3.2. Baseline Demographic Characteristics of 327 Patients

Patients’ baseline demographic characteristics are shown in Table 1. Patients were predominantly middle-aged (mean age, 57.1 ± 13.6 years) and male (72%). Evidence of chronic viral hepatitis infection was seen in 58.7% of patients. Various abnormal laboratory data could be interpreted as follows: renal function impairment (creatinine 1.2 (0.9–2.4) mg/dL), hyponatremia (sodium 135 (131–139) mg/dL), hyperbilirubinemia (bilirubin 4.1 (1.9–9.8) mg/dL), hypoalbuminemia (albumin 2.4 (2.2–2.8) g/dL), the prolonged international normalized ratio for the prothrombin time (INR 1.6 (1.4–2.2)), anemia (Hb 9.6 (8.4–11.0) g/dL), and thrombocytopenia (PLT 74.0 (48.0–122.0) × 1000/μL). The median WBC counts of blood were 7.9 (5.2–12.8) × 1000 per mL. Anti-viral agents included Entecavir 19.5% (64), Lamivudine 1.2% (4), Telbivudine 0.6% (2), and Tenofovir disoproxil fumarate 4.5% (15).

### 3.3. Baseline Characteristics and Prognosis Comparison of the Three Types of SBP 

There were three types of SBP as shown in Table 1 and Table 2. The number of patients with classic SBP was 141 (43.2%). A total of 143 patients (43.7%) were CNNA. Moreover, there were 43 patients (13.1%) with MNB. Furthermore, 42 (12.8%) patients showed both positive ascites and blood culture, defined as bacteremia at diagnosis (Table 1). There was no significant difference in age, sex, etiology, baseline MELD score, CTP score, INR, WBC, Hb, PLT, and serum bilirubin total among the three types of SBP. In contrast, there were significant differences in bacteremia rate, iMELD score, creatinine, serum sodium, and albumin among the three types of SBP.

The mortality rate among the three types of SBP were further compared. There was no significant difference in the in-hospital, 3-month, and 6-month mortality rates among the three types of SBP (*p*-value 0.841, 0.461, 0.951, respectively).

### 3.4. The Bacteriology of SBP

**Figure 2:**
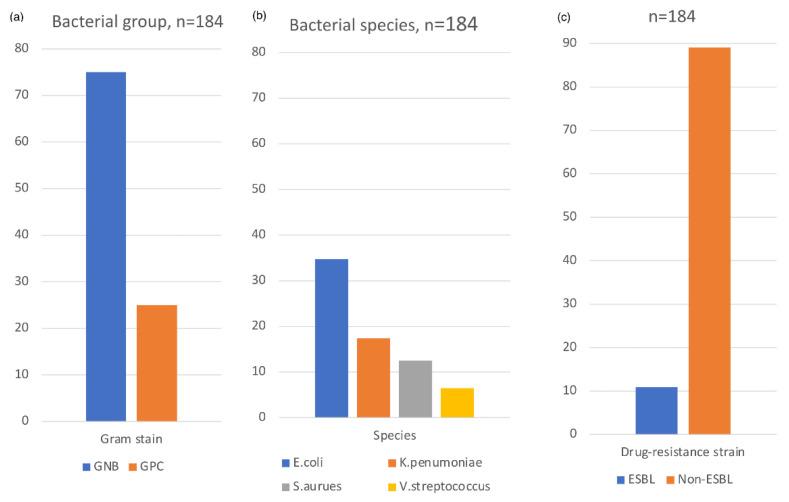
Schematic demonstration of the bacteriology of SBP. (**a**) GNB (75%) vs. GPC (25%) (**b**) Overall, *E. coli* was the most common bacteria species. (**c**) The ESBL strain in all culture-positive patients was 10.9%.

In addition, the bacteriology of SBP was studied. First, ascites analysis revealed a median WBC of 1585 (IQR: 439–4996), median ascites PMN of 1394 (344–4203) cells/mm^3^, ascites total protein of 1.3 (1.3–1.7) g/dL, and ascites albumin of 0.5 (0.3–0.7) g/dL (Table 3). Second, as shown in Table 3 and Figure 2a, among those 184 patients with positive bacterial culture results, 138 patients (75.0%) yielded Gram-negative bacteria (GNB). Among them, 110 patients (79.7%) yielded Ceftriaxone-sensitive GNB while 28 patients (20.3%) yielded Ceftriaxone-resistant GNB in their cultures. On the other hand, 46 (25.0%) of the 184 culture-positive patients yielded Gram-positive coccus (GPC).

Overall, the most common bacteria species was *Escherichia coli* (64 patients,34.8%), of which 14 patients (21.8%) had extended-spectrum beta-lactamases (ESBL) (Table 3 and Figure 2b). The second most common was *Klebsiella pneumoniae* (32 patients, 17.4%), of which only one (3.1%) had ESBL. *Staphylococcus aureus* was the most common GPC (23 patients, 12.5%), of which 5 patients (21.7%) were ORSA. *Viridans streptococcus* was the second most common GPC (12 patients, 6.5%). The total percentage of ESBL strain in culture-positive patients was 10.9% (20 over 184 patients), as revealed in Figure 2c.

There was no significant etiologic difference (etiology of cirrhosis) between the classic SBP and MNB (*p* = 0.065). However, there were significant differences in bacteriology between the classic SBP and MNB (Table 4).

### 3.5. Multivariable Logistic Regression Analysis to Predict Mortality

Furthermore, univariable then multivariable logistic regression analyses were performed to analyze independent factors that predict in-hospital, 3-month, and 6-month mortality.

#### 3.5.1. In-Hospital Mortality

As shown in Table 5, univariable and multivariable logistic regression analysis for predicting in-hospital mortality confirmed that SBP type did not affect in-hospital mortality. Neither Gram stain types nor ESBL strain in bacterial culture affected the in-hospital mortality. Age, sex, etiology, hepatic encephalopathy (HE), serum albumin, CTP score, and MELD-based scores had no significant effect, either. In contrast, bacteremia, 3rd-generation cephalosporin (CRO)-resistant bacteria in ascites culture, hepatorenal syndrome, and serum creatinine were independent predictors of patients’ in-hospital mortality (Table 5).

#### 3.5.2. 3-Month Mortality

As shown in Table 6, univariable and multivariable logistic regression analysis for predicting 3-month mortality confirmed that SBP type did not affect in-hospital mortality. Gram stain types, ESBL strain, or CRO-resistant strain in bacterial culture did not affect the 3-month mortality. Age, sex, etiology, hepatic encephalopathy (HE), serum albumin, CTP score, and MELD-based scores had no significant effect, either. In contrast, bacteremia, hepatorenal syndrome, and serum creatinine were independent predictors of patients’ 3-month mortality (Table 6).

#### 3.5.3. 6-Month Mortality

As shown in Table 7, univariable and multivariable logistic regression analysis for predicting 6-month mortality confirmed that SBP type did not affect in-hospital mortality. Bacteremia, Gram stain types, ESBL strain, or CRO-resistant strain in bacterial culture did not affect the 6-month mortality. Age, sex, etiology, hepatic encephalopathy (HE), serum albumin, CTP score, and MELD-based scores had no significant effect, either. In contrast, hepatorenal syndrome and serum creatinine were independent predictors of patients’ 3-month mortality (Table 7).

## 4. Discussion

In this study, we attempted to find better prognostic risk factors for cirrhotic patients with SBP, taking into account all clinical variables, including SBP types, bacteriology, and other cirrhotic complications such as HRS and HE. It was found that the prevalence of classic SBP was nearly equivalent to CNNA, followed by MNB. As for the microbial pattern, GNB was still more prevalent than GPC (75% vs. 25%), and *E. coli* were the most common bacteria species followed by *K.P*. and then *Staphylococcus*. The total percentage of ESBL strain in culture-positive patients was 10.9%. By univariable and multivariable logistic regression survival analysis, there was no significant difference in predicting short-term mortality among the three SBP types, neither between GNB vs. GPC, nor between ESBL- and non-ESBL- producing bacteria. Only bacteremia (sepsis), hepatorenal syndrome (HRS), and serum creatinine (Cr) were independent predictors of in-hospital and 3-month mortality, whereas HRS and Cr were independent predictors of 6-month mortality. The results could greatly help identify high-risk groups of patients with SBP, allowing more prompt and intensive management.

SBP has high short-term mortality. When first reported, the in-hospital mortality of an episode of SBP exceeded 90%; however, the rate has been reduced to approximately 20% through early diagnosis and prompt antibiotic therapy [27,28]. To improve the stratification of patient care, identifying the most robust predictors of mortality in cirrhotic patients with SBP is critical but often overlooked [29]. The MELD score has been shown to be more accurate in predicting 3-month survival than the Child−Turcotte−Pugh (CTP) classification for patients with cirrhosis awaiting liver transplantation in the United States [30]. However, the literature review showed limited information on whether they were applicable in subgroups of patients with liver cirrhotic-related complications such as SBP [31,32].

We have previously demonstrated that for patients with HBV-related liver cirrhosis and SBP, the iMELD score had the highest AUC among the MEDL-based models and significantly outperformed CTP and ALBI scores in predicting 3-month and 6-month mortalities [33]. However, baseline clinical parameters such as SBP types, bacteriology, HRS, and HE were not considered. In this study, univariable and multivariable logistic regression survival analyses were used to consider all these variables and MELD-based scores, including the iMELD score. The results showed that only HRS and serum Cr consistently predicted the in-hospital, 3-month, and 6-month mortalities. This corresponds to a meta-analysis that also demonstrated that renal dysfunction was the most important independent predictor of mortality in cirrhotic patients with SBP [29]. In fact, renal failure occurs in 30% to 40% of people with SBP and is the leading cause of death [34]. The risk of renal impairment as well as mortality may be decreased significantly (renal impairment 30.6% to 8.3%, mortality 35.4% to 16.0%) [35] with an infusion of intravenous 25% albumin solution [36]. Therefore, the up-to-date AASLD guideline has recommended albumin infusion in patients with SBP and renal dysfunction [20]. Our result strengthens this notion that early identification of renal dysfunction at baseline in patients with SBP is critical and potentially life-saving.

The study also demonstrated that bacteremia (sepsis) is an important prognostic factor in predicting in-hospital and 3-month mortalities. Our previous study also found that SBP was associated with high sepsis-related mortality [37]. This implies that aggressive treatment for sepsis in patients with SBP is of utmost importance [38]. Indeed, an important predictive scoring system designed to assess the severity of illness in patients with sepsis, the Chronic Liver Failure-Sequential Organ Failure Assessment (CLIF-SOFA) score, has been shown to be useful in determining the appropriate antibiotic regimen [39]. Patients with suspected SBP who are not critically ill (CLIF-SOFA score < 7) are typically treated with a third-generation cephalosporin. Conversely, for patients with a CLIF-SOFA score ≥ 7, empiric treatment with carbapenems is recommended [39].

Furthermore, in this study, the prevalence of classic SBP was almost comparable to that of CNNA, which corresponds to other studies [10,40]. The mortality rates among these classic, CNNA, MNB SBP types were not significantly different if appropriate antibiotics are given promptly. In addition, GNB vs. GPC, nor ESBL-producing vs. non-ESBL-producing bacterial species did not affect outcomes. This finding is reasonable since empirical 3rd generation cephalosporins could cover 79.7% of patients without ESBL-producing bacteria strain. The finding that third generation cephalosporin (CRO)-resistant bacteria in ascites culture independently predicted patients’ in-hospital mortality reminds us of the need for timely antibiotic adjustment based on the susceptibility results.

Thus, liver transplantation should be seriously considered for survivors of SBP who are otherwise good transplantation candidates [35].

## 5. Conclusions

The short-term mortality rate of SBP remains high. By multivariable logistic regression analysis, there was no significant difference in predicting short-term mortality among the three SBP types, neither between GNB vs. GPC nor between ESBL- and non-ESBL-producing bacteria. Only bacteremia (sepsis), hepatorenal syndrome (HRS), and serum creatinine (Cr) were independent predictors of in-hospital and 3-month mortality, whereas HRS and Cr were independent predictors of 6-month mortality. The results could greatly help identify high-risk groups of patients with SBP, allowing more prompt and intensive management such as albumin infusion or liver transplantation.

## Figures and Tables

**Table 1:** Demographic and baseline clinical characteristics of 327 patients with the first episode of SBP.

Baseline Parameters	Values
Clinical parameters	
Age, mean ± SD	57.1 ± 13.6
Male No. (%)	236 (72%)
Etiology No. (%)	
Alcohol HBV HCV Others	94 (28.7) 118 (36.0) 74 (22.6) 74 (22.6)
Classic (Positive PMN and culture)	141 (43.2)
CNNA	143 (43.7)
MNB	43 (13.1)
Blood culture positive	42(12.8%)
Laboratory parametersMedian (IQR)	
iMELD score	44.44 (37.58–52.94)
MELD score	21.11 (15.39–28.36)
CTP score	9(8–11)
INR	1.64 (1.39–2.20)
WBC (10^3^/μL)	7.9 (5.20–12.80)
Hemoglobin (g/dL)	9.6 (8.4–11.0)
PLT (10^3^/μL)	74.0 (48.0–122.0)
Creatinine (mg/dL)	1.2 (0.86–2.41)
Bilirubin Total (mg/dL)	4.1 (1.9–9.8)
AST (U/L)	70 (46–129)
ALT (U/L)	36 (23–60)
Sodium (mEq/L)	135 (131–139)
Albumin (g/dL)	2.4 (2.2–2.8)
Anti-viral agents, n, (%)	
Entecavir	64(19.5)
Lamivudine	4 (1.2)
Telbivudine	2 (0.6)
Tenofovir disoproxil fumarate	15 (4.5)

CNNA: culture-negative neutrophilic ascites; MNB: monomicrobial non-neutrocytic bacterascites; iMELD: integrated MELD score.

**Table 2:** Demographic and baseline clinical characteristics of three types of SBP.

SBP Types(n)	Classic (141)	CNNA (143)	MNB (43)	*p*-Value
Clinical parameters				
Age, mean ± SD	57.6 ± 14.1	57.3 ± 13.5	55.1 ± 12.8	0.578
Male No. (%)	104(73.8)	101(70.6)	31(72.1)	0.841
Etiology No. (%)				0.157
Alcohol HBV HCV Others	43(30.5) 58(40.2) 36(25.5) 4(2.8)	39(27.3) 65(45.5) 33(23.0) 6(4.2)	10(23.3) 23(53.5) 9(20.9) 1(2.3)	
Blood culture positive, n (%)	39(27.7)	0	3(7)	<0.001
Laboratory parametersMedian (IQR)				
iMELD score	44.8(30.7–55.0)	44.6(37.4–53.2)	40.2(33.9–47.5)	0.015
MELD score	20.5(15.8–28.6)	21.9(12.6–30.5)	21.2(12.6–30.5)	0.055
CTP score	9(8–10)	10(8–11)	9(8–11)	0.196
INR	1.7(1.4–2.1)	1.6(1.4–2.3)	1.6(1.3–2.2)	0.869
WBC (103/μL)	8.4(5.8–11.6)	7.8(5.5–13.3)	6.6(4.0–12.6)	0.348
Hemoglobin (g/dL)	9.6(8.4–11.2)	9.8(8.5–11.1)	9.2(8.1–10.2)	0.248
PLT (103/μL)	72(46–105)	76(50–139)	72(46–120)	0.259
Creatinine (mg/dL)	1.5(1.0–2.7)	1.0(0.7–1.8)	1.0(0.7–2.0)	<0.001
Bilirubin Total (mg/dL)	4.0(2.1–8.8)	4.2(2.0–11.0)	3.0(1.4–7.8)	0.457
Sodium (mEq/L)	135(130–138)	134(131–139)	137(135–141)	<0.001
Albumin (g/dL)	2.3(2.0–2.6)	2.6(2.3–3.0)	2.4(2.1–2.8)	<0.001

CNNA: culture-negative neutrophilic ascites; MNB: monomicrobial non-neutrocytic bacterascites; iMELD: integrated MELD score.

**Table 3:** Bacterial culture results of 327 patients with first SBP episode.

Ascites analysis, (Median (IQR))	
WBC (10^3^/μL)	1585 (439–4996)
PMN (cells/mm^3^)	1394 (344–4203)
Total protein (g/dL)	1.3 (1.3–1.7)
Albumin (g/dL)	0.5 (0.3–0.7)
Bacterial group, n, (%), total *n* = 184	
Gram-negative bacteria (GNB)	138 (75.0)
Ceftriaxone sensitive GNB	110 (79.7)
Ceftriaxone resistant GNB	28 (20.3)
Gram-positive coccus (GPC)	46 (25.0)
Bacterial species, n, (%), total *n* = 184	
*Escherichia coli*	64 (34.8)
*Escherichia coli*, ESBL strain	14/64 (21.8)
*Klebsiella pneumoniae*	32 (17.4)
*Klebsiella pneumoniae*, ESBL strain	1/32 (3.1)
*Staphylococcus aureus*	23 (12.5)
ORSA	5/23 (21.7)
*Viridans streptococcus*	12 (6.5)

ESBL: extended-spectrum beta-lactamases; ORSA: Oxacillin-resistant Staphylococcus aureus.

**Table 4:** Bacterial culture differences between classical SBP and MNB.

SBP Types(n)	Classic (141)	MNB (43)	*p*-Value
GNB vs. GPC			0.005
GNB, No. (%)	110(78)	29(67.4)	
GPC, No. (%)	34 (22)	14(32.6)	
Ceftriaxone sensitive vs. resistant GNB			
Ceftriaxone sensitive GNB	91(82.7)	19(65.5)	0.008
Ceftriaxone resistant GNB	19(17.3)	9(34.5)	0.233
ESBL strain, No. (%)	15(13.6)	0(0)	<0.001

**Table 5:** Univariable and multivariable logistic regression analysis for predicting in-hospital mortality.

	Univariable Logistic Reg		Multivariable Logistic Reg	
Variables	OR (95% CI)	*p*-Value	OR (95% CI)	*p*-Value
SBP types				
CNNA	Reference			
MNB	0.938 (0.459–1.914)	0.859		
Classic	1.373 (0.853–2.211)	0.191		
Bacteremia	2.286 (1.185–4.408)	0.014	3.192 (1.419–7.176)	0.005
Gram stain				
0	Reference			
GNB	1.283 (0.796–2.066)	0.306		
GPC	1.391 (0.690–2.805)	0.356		
ESBL strain	2.366 (0.654–8.553)	0.189		
CRO-resistant				
0	Reference		Reference	
Ascites	5.155 (1.623–16.373)	0.005	6.493 (1.791–23.538)	0.004
Blood cult.	1.145 (0.188–6.961)	0.883	1.487 (0.232–9.515	0.676
Ascites + Blood	4.295 (0.82–22.514)	0.085	0.803 (0.113–5.720)	0.826
Age	1.018 (1.001–1.035)	0.038	1.013 (0.992–1.034)	0.242
Sex	0.875 (0.535–1.432)	0.596		
Etiology				
Alcohol	Reference			
HBV	1.117 (0.659–1.891)	0.682	1.196 (0.638–2.244)	0.576
HCV	0.429 (0.221–0.833)	0.012	0.582 (0.265–1.277)	0.177
Others	0.762 (0.208–2.787)	0.681	0.746 (0.175–3.337)	0.720
HE				
0	Reference		Reference	
1	1.538 (0.706–3.349)	0.278	1.161 (0.436–3.094)	0.765
2	2.766 (1.302–5.876)	0.008	1.862 (0.764–4.538)	0.171
3–4	3.786 (1.253–11.437)	0.018	1.843 (0.537–6.330)	0.331
HRS	9.744 (3.633–26.132)	<0.001	7.274 (2.497–21.192)	<0.001
Cr	1.397 (1.218–1.601)	<0.001	1.198 (1.035–1.388)	0.015
Albumin	0.836 (0.536–1.305)	0.431		
WBC	1.031 (1.004–1.058)	0.023	1.026 (1–1.052)	0.049
CTP score	1.257 (1.091–1.448)	0.002	1.118 (0.899–1.391)	0.316
MELD score	1.05 (1.026–1.075)	<0.001	0.985 (0.926–1.047)	0.622
MELD-Na	1.048 (1.025–1.071)	<0.001	1.04 (0.98–1.094)	0.126
iMELD score	1.05 (1.028–1.073)	<0.001	1.009 (0.948–1.073)	0.783

CNNA: culture-negative neutrophilic ascites; MNB: monomicrobial non-neutrocytic bacterascites; GNB: Gram-negative bacillus; GPC: Gram-positive coccus; ESBL: extended-spectrum beta-lactamases; HE: hepatic encephalopathy; HRS: hepatorenal syndrome; iMELD: integrated MELD score.

**Table 6:** Univariable and multivariable logistic regression analysis for predicting 3-month mortality.

	Univariable Logistic Reg		Multivariable Logistic Reg	
Variables	OR (95% CI)	*p*-Value	OR (95% CI)	*p*-Value
SBP types				
CNNA	Reference			
MNB	0.938 (0.459–1.914)	0.859		
Classic	1.328 (0.833–2.117)	0.234		
Bacteremia	2.343 (1.170–4.691)	0.016	2.244 (1.077–4.673)	0.031
Gram stain				
0	Reference			
GNB	1.328 (0.834–2.115)	0.232		
GPC	1.422 (0.709–2.849)	0.321		
ESBL strain	2.261 (0.574–8.900)	0.243		
CRO-resistant				
0	Reference			
Ascites	3.102 (0.978–9.837)	0.055		
Blood cult.	1.551 (0.255–9.416)	0.883		
Ascites + Blood	6.204 (0.738–52.16)	0.093		
Age	1.010 (0.994–1.026)	0.24		
Sex	1.113 (0.686–1.805)	0.665		
Etiology				
Alcohol	Reference			
HBV	0.775 (0.456–1.315)	0.344		
HCV	0.452 (0.244–0.838)	0.012		
Others	0.571 (0.162–2.010)	0.383		
HE				
0	Reference			
1	1.325 (0.611–2.874)	0.476		
2	2.369 (1.077–5.214)	0.032		
3–4	2.154 (0.715–6.487)	0.173		
HRS	7.420 (2.534–21.732)	<0.001	6.034 (1.977–18.416)	0.002
Cr	1.283 (1.122–1.468)	<0.001	1.188 (1.024–1.378)	0.023
Albumin	0.643 (0.414–1.000)	0.431	0.711 (0.436–1.157)	0.170
WBC	1.017 (0.994–1.041)	0.158		
CTP Score	1.169 (1.020–1.340)	0.025	1.128 (0.929–1.370)	0.225
MELD score	1.033 (1.010–1.056)	0.005	0.968 (0.918–1.020)	0.221
MELD-Na	1.029 (1.009–1.051)	0.006	0.995 (0.953–1.039)	0.811
iMELD score	1.035 (1.014–1.057)	0.001	1.040 (0.991–1.092)	0.107

CNNA: culture-negative neutrophilic ascites; MNB: monomicrobial non-neutrocytic bacterascites; GNB: Gram-negative bacillus; GPC: Gram-positive coccus; ESBL: extended-spectrum beta-lactamases; HE: hepatic encephalopathy; HRS: hepatorenal syndrome; iMELD: integrated MELD score.

**Table 7:** Univariable and multivariable logistic regression analysis for predicting 6-month mortality.

	Univariable Logistic Reg		Multivariable Logistic Reg	
Variables	OR (95% CI)	*p*-Value	OR (95% CI)	*p*-Value
SBP types				
CNNA	Reference			
MNB	1.118 (0.554–2.259)	0.755		
Classic	1.036 (0.644–1.668)	0.883		
Bacteremia	1.963 (0.948–4.061)	0.069	1.797 (0.829–3.894)	0.138
Gram stain				
0	Reference			
GNB	0.982 (0.612–1.576)	0.939		
GPC	1.436 (0.689–2.993)	0.334		
ESBL strain	1.519 (0.386–5.985)	0.550		
CRO-resistant				
0	Reference			
Ascites	2.039 (0.643–6.472)	0.227		
Blood cult.	1.020 (0.168–6.194)	0.983		
Ascites + Blood	4.079 (0.485–34.306)	0.196		
Age	1.015 (0.999–1.032)	0.07	1.009 (0.989–1.030)	0.388
Sex	1.239 (0.758–2.025)	0.393		
Etiology				
Alcohol	Reference			
HBV	0.856 (0.497–1.474)	0.576		
HCV	0.633 (0.341–1.175)	0.147		
Others	1.446 (0.358–5.835)	0.604		
HE				
0	Reference			
1	1.329 (0.593–2.978)	0.490		
2	1.538 (0.698–3.390)	0.285		
3–4	2.797 (0.769–10.169)	0.118		
HRS	4.866 (1.661–14.260)	0.004	3.857 (1.248–11.919)	0.019
Cr	1.343 (1.146–1.574)	<0.001	1.218 (1.030–1.441)	0.021
Albumin	0.685 (0.438–1.070)	0.097	0.731 (0.443–1.204)	0.218
WBC	1.035 (1.002–1.069)	0.035	1.029 (0.996–1.062)	0.083
CTP Score	1.142 (0.993–1.313)	0.062	1.126 (0.924–1.373)	0.240
MELD score	1.030 (1.006–1.055)	0.013	0.972 (0.918–1.028)	0.316
MELD-Na	1.024 (1.003–1.045)	0.024	0.982 (0.967–1.028)	0.437
iMELD score	1.036 (1.014–1.058)	0.001	1.048 (0.991–1.107)	0.098

## Data Availability

Data and study materials will be made available to other researchers by email request.
